# Optimisation of Almond-Based Dairy-Free Milk Alternative Formulation Fortified with Myrtle, Bay Leaf and Fennel Extracts^§^

**DOI:** 10.17113/ftb.61.03.23.8002

**Published:** 2023-09

**Authors:** Sandra Balbino, Daniela Cvitković, Hanna Skendrović, Verica Dragović-Uzelac

**Affiliations:** Faculty of Food Technology and Biotechnology, University of Zagreb, Pierottijeva 6, 10000 Zagreb, Croatia

**Keywords:** almond drink, herbal extract, antioxidant activity, phenols, sensory analysis

## Abstract

**Research background:**

Herbs and spices used in traditional medicine are nowadays increasingly used in combinations to create functional food formulations aimed at treating specific symptoms and disorders. Among herbs originating from the Mediterranean region, extracts of myrtle (*Myrtus communis* L.), bay leaf (*Laurel nobilis* L.) and fennel (*Foeniculum vulgare* Mill.) are traditionally used for gastrointestinal disorders. When considering how to incorporate these extracts into products, dairy-free milk alternatives provide an excellent base with almond-based drinks being among the most popular within this group.

**Experimental approach:**

The aim of this study is therefore to optimise the formulation of an almond drink fortified with a 25 % (on dry mass basis) aqueous herbal extract containing myrtle, bay leaf (25 % each) and fennel seed (50 %) extracts. A central composite design with 20 formulations varied the content of *φ*(aqueous herbal extract)=2–6 %, lecithin as emulsifier 0.15–0.45 and xylitol as sweetener 2–5 % (*m*/*V*), while antioxidant activity, total phenolic content and sensory properties were determined as dependent variables.

**Results and conclusions:**

The antioxidant activity and total phenolic content of the prepared almond drink formulations increased with the amount of added concentrated aqueous herbal extracts, as did the colour, herbal odour and flavour, bitterness and aftertaste. The addition of lecithin resulted in a darker colour and the sweetness was increased by the xylitol content. All formulations had good overall attractiveness, which increased with higher xylitol content.

**Novelty and scientific contribution:**

The current work offers new insights into the optimisation of fortified dairy-free alternatives. The addition of myrtle, bay leaf and fennel seed extracts to almond drink-based formulations resulted in a 12-fold increase in antioxidant activity. Xylitol masks the potential bitterness of the phenolic compounds so that higher amounts of extracts can be added.

## INTRODUCTION

Considering modern trends in functional nutrition, herbal extracts from spices and aromatic plants known in traditional medicine are increasingly used in the preparation of functional food formulations. Their medicinal effects include antioxidant, antimicrobial, anti-inflammatory, anticarcinogenic activities and other specific activities in prophylaxis and treatment of various systematic disorders associated with different phytochemicals (*1*). Phenolic compounds are one of the most abundant groups of bioactive phytochemicals found in herbs and aromatic plants as secondary metabolites and are involved in defence mechanisms against biotic and abiotic stress. The main subgroups with pronounced bioactive effects include phenolic acids and flavonoids.

The almond drink originated in the Mediterranean region and has been used since the Middle Ages, when it was one of the staple foods because cow's milk did not have a long shelf life due to spoilage (*2*). The almond (*Prunus dulcis* L.), a member of the genus *Prunus* in the Rosaceae family, is native to Central Asia and is cultivated in Mediterranean climates, including California and Australia (*3*).

Recently, the demand for non-dairy milk alternatives has increased due to allergies to milk proteins and lactose, and almond drinks have received much attention due to their good nutritional value and functionality (*4*). The global almond drink production industry is growing every year and is expected to grow by 8.8 % per year from 2021 to 2031 (*5*).

Almond drink does not contain lactose and is therefore suitable for people with lactose intolerance or milk protein allergies. It can also be a suitable substitute for cow's milk for vegans and adult vegetarians (*2*). Because it does not contain high amounts of saturated fat and is low in calories, it can also be of interest to individuals who want to limit their consumption of saturated fat. In addition, almonds are an excellent source of several bioactive compounds such as α-tocopherol and manganese (*2*, *6*). They also have a low glycaemic index and do not negatively affect insulin sensitivity (*7*). In addition to all these benefits, the pectin substances in the cell wall help to reduce the amount of low-density lipoprotein (LDL) cholesterol in plasma (*6*, *8*). Almonds are also known for their gastrointestinal activity and potential prebiotic properties due to arabinose, while their consumption increases butyrate amounts, which may be associated with a beneficial effect on the functionality of the microbiota. They are also considered to have a significant inhibitory effect on peptic activity, as their consumption increases the pH of gastric juice and reduces the production of hydrochloric acid (*9*).

During the production of the almond drinks, the almonds are soaked, then drained and rinsed with cold water (*4*). The next step is blanching, followed by wet grinding, where water is added to the raw material (*10*) together with an emulsifier (*11*). Large particles are then removed by filtration or centrifugation (*6*). Homogenisation is then carried out, which reduces the size of the fat globules and results in a uniform texture and appearance of the beverage (*2*). Due to its excellent sensory properties, almond drink has recently become increasingly popular among consumers of dairy-free milk alternatives. Compared to soy drink, it has a higher ash content due to higher amounts of calcium, potassium, phosphorus, magnesium, iron and zinc, as well as a higher crude fibre content (*12*). A soy and almond milk alternative with almond content of 60 or 40 % was also compared to regular milk and it had higher nutrient concentrations except for calcium and protein (*13*). A study by Al Tamimi (*14*) also showed that replacing dairy products with almond drinks can lead to a reduction in body mass index.

Since almond drink is a highly nutritious medium, it is susceptible to spoilage and the development of pathogenic microorganisms. Therefore, almond drink undergoes heat treatment to prolong its microbiological stability and also inactivate lipases that can lead to its hydrolytic deterioration (*15*). It also contains high concentrations of polyunsaturated fatty acids, which are susceptible to oxidative degradation (*16*). To slow down oxidation, improve sensory properties and add additional functionality, the almond drink can be fortified with herbal extracts. Furthermore, its mentioned biological activity can be improved by adding a combination of extracts of various aromatic herbs known for their specific medicinal effects.

For this study, myrtle, bay leaf and fennel were selected as herbs with known gastrointestinal effects, which can thus complement the bioactivity of almonds. Myrtle (*Myrtus communis* L.) is an evergreen shrub of the myrtle family (Myrtaceae) that is widely distributed in the Mediterranean region. In addition to digestive disorders, the plant is traditionally used to treat urinary tract infections, bronchial congestion, sinusitis and dry cough (*17*). *Laurus nobilis* L., better known as bay leaf, belongs to the Lauraceae family and is one of the most commonly used culinary spices in many countries (*18*). Laurel originates from southwestern Asia and southeastern Europe and has spread throughout the Mediterranean region (*19*). The bay leaves are also traditionally used to treat various gastrointestinal symptoms such as diarrhoea, epigastric bloating and flatulence (*20*). Both myrtle and bay leaves are rich in various bioactive compounds such as phenols, carotenoids and chlorophylls (*21*, *22*). Fennel (*Foeniculum vulgare* Mill.) is one of the ancient herbs. It belongs to the Apiaceae family and is native to the Mediterranean region (*23*). It is effective in treating gastrointestinal complaints such as constipation, diarrhoea, flatulence, gastralgia, gastritis, irritable bowel syndrome, stomach pain, and as an anticolic and laxative agent. It has been shown to have a beneficial effect on inflammatory bowel disease (*24*).

Although sensory attributes are not the main reason for consuming functional foods and beverages, they can have a major impact on the appeal of the product to consumers (*25*). For functional foods produced by the addition of herbal extracts, sensory properties can be influenced by specific herbal aromas or high contents of phenolic compounds. In particular, phenolic compounds are known for their bitter taste, which can lead to consumer acceptance problems (*26*). Therefore, not only bioactivity but also product acceptance must be considered when optimising formulations. Thus, the aim of this study is to develop an optimal formulation for an almond drink fortified with myrtle, bay leaf and fennel extracts and to determine the influence of the amount of extract, emulsifier and sweetener on the content of phenolic compounds, antioxidant activity and sensory properties. In addition, the results of this study could provide an optimisation procedure for the development of other dairy-free milk alternatives enriched with herbal extracts.

## MATERIALS AND METHODS

### Preparation of the basic almond drink

The basic almond drink was prepared from commercially available shelled almonds (Lidl Ltd, Neckarsulm, Germany) by soaking them overnight in water at a ratio of 1:3. The almonds were then rinsed and blanched in hot water (95 °C) to facilitate removal of the skin. The peeled almonds were mixed with water in a ratio of 1:9 and the mixture was ground in a blender (HR2052/00; Philips, Amsterdam, Netherlands). The resulting mass was filtered through a cheesecloth and filter paper. The filtered basic almond drink was placed in a glass bottle and stored in the refrigerator (4 °C).

### Preparation of concentrated aqueous herbal extract

The extracts of myrtle, bay leaves and fennel seeds were prepared by weighing 30 g of dry plant material and twofold extracting with 200 mL of *φ*(ethanol)=30 % for 10 min in a water bath with a shaker (Cole-Parmer™ Stuart™ SBS40; Cole-Parmer, Antylia Scientific Company, St Neots, UK) at 60 °C. The obtained extracts were filtered and centrifuged at 4752×*g* for 10 min (Hettich, Tuttlingen, Germany). They were then concentrated (Heidolph Instruments GmbH & Co. KG, Schwabach, Germany) to a dry matter content of 25 % with the mixture containing 25 % myrtle leaf extract, 25 % bay leaf extract and 50 % fennel seed extract.

### Preparation of formulations

The experimental design included 20 almond drink formulations containing different amounts of concentrated aqueous herbal extract, xylitol (Lidl Ltd, Neckarsulm, Germany) and lecithin (Miovolis, Karlsruhe, Germany). Deoiled lecithin granules were homogenised in a small quantity of basic almond drink in an ultrasonic bath to dissolve the lecithin before the addition to the formulation. Prepared formulations were subjected to homogenisation (T 25 Ultra Turrax; IKA, Staufen, Germany) for 5 min at 10 000 rpm. They were then pasteurised at 72 °C for 15 s. The pasteurised samples were transferred into pre-sterilised plastic vials immediately after pasteurisation. The samples were stored in a refrigerator (4 °C) until analysis.

**Fig. 1:**
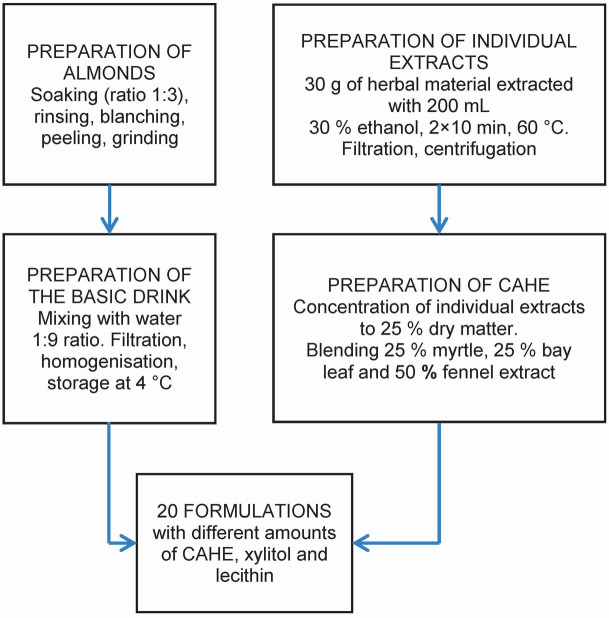
Preparation scheme of fortified almond drink formulations containing different amounts of concentrated aqueous herbal extract (CAHE), xylitol and lecithin

Overall process of preparation of fortified almond drink formulations is shown in Fig. 1.

### Reagents and materials

Ethanol (96 % p.a.), sodium acetate (anhydrous, p.a.), acetic acid (glacial, p.a.) and sodium carbonate (anhydrous, p.a.) were obtained from Gram-Mol (Zagreb, Croatia). Folin-Ciocalteu reagent was purchased from Reagecon (Shannon, Ireland) while gallic acid, iron(III) chloride hexahydrate (≥98 %), 2,4,6-Tris(2-pyridyl)-*s*-triazine and Trolox (97 %) standards were from Sigma-Aldrich, Merck (St. Louis, MO, USA).

### Determination of total phenols

The spectrophotometric method based on the colorimetric reaction between the Folin-Ciocalteu reagent and phenolic compounds as the reducing reagent was used to determine the total phenolic content (TPC) (*27*). A volume of 100 μL sample, 200 μL Folin-Ciocalteu reagent and 2 mL distilled water were pipetted into a glass test tube and after 3 min saturated sodium carbonate solution (1 mL) was added. After incubation at 50 °C for 25 min, the resulting absorbance was measured at 765 nm against a blank sample prepared in the same way, with distilled water added instead of the sample. The samples were calibrated with 50, 100, 150, 250 and 500 mg/L gallic acid solution.

### Determination of antioxidant activity

The Fe(III) reducing antioxidant power (FRAP) method was used to determine antioxidant activity (*28*). The method is based on the reduction reaction of the yellow-coloured complex iron-2,4,6-tripyridyl-*s*-triazine (TPTZ) in an acidic medium, forming a blue-coloured iron(II)-tripyridyltriazine complex, which has an absorption maximum at 593 nm. A volume of 240 μL distilled water, 80 μL sample and 2080 μL FRAP reagent were added to the glass tubes, vortexed at 1800 rpm (IKA MS 3; IKA) and incubated at 37 °C for 5 min. Then the absorbance was measured at 593 nm against a blank sample containing everything except the sample, with the distilled water added instead. FRAP values were expressed as 6-hydroxy-2,5,7,8-tetramethylchroman-2-carboxylic acid (Trolox) equivalents (TE), for which calibration was made in the concentrations of 25, 100, 125, 250, 500 and 1000 µmol/L. The results were calculated and expressed as Trolox equivalents in µmol/L.

### Sensory evaluation

Sensory analysis was performed with 10 panellists (9 female and 1 male, 30–60 years old). Each sensory property of the sample was assigned a numerical score ​​from 1 to 10. A score of 1 indicated the absence of the evaluated property (unexpressed property), and a score of 10 indicated the most intense property of the sample. The colour intensity was tested in relation to the colour of the basic drink, which corresponded to a score of 1. The homogeneity, herbal odour and flavour, sweetness, bitterness, aftertaste and overall acceptability of the samples were also evaluated. Finally, the overall acceptability of the sample was examined and also given a score from 1 to 10.

### Experimental design and statistical analysis

The Design-Expert 10.0 software (*29*) was used for experimental design and statistical data processing using the Box-Wilson design. In Box-Wilson central composite design (CCD) each factor is varied at five levels, but not all combinations of levels are used. The amounts of concentrated aqueous herbal extract and lecithin and xylitol in the formulations were chosen as independent factors and each factor was tested at five levels (-α, -1, 0, +1 and +α) and the central point was tested in 5 repetitions, resulting in a total of 20 trials. The levels for the independent variables were selected based on preliminary experiments (data not shown) as follows: (*i*) the volume fraction of added concentrated aqueous herbal extract 2 to 6 %, (*ii*) the amount of lecithin 0.15 to 0.45 % (*m*/*V*) and (*iii*) the amount of xylitol 2 to 5 % (*m*/*V*), as shown in Table 1.

**Table 1:** Experimental design of fortified almond drink formulations with different amounts of concentrated aqueous herbal extract (CAHE), lecithin and xylitol

Formulation	A *φ*(CAHE)/%	B (*m*(lecithin)/*V*(sample))/%	C (*m*(xylitol)/*V*(sample))/%
1	0.64	0.30	3.50
2	4.00	0.30	3.50
3	4.00	0.30	6.02
4	2.00	0.15	2.00
5	4.00	0.30	0.98
6	7.36	0.30	3.50
7	6.00	0.45	5.00
8	4.00	0.30	3.50
9	6.00	0.15	2.00
10	4.00	0.30	3.50
11	2.00	0.45	2.00
12	6.00	0.45	2.00
13	4.00	0.30	3.50
14	4.00	0.05	3.50
15	6.00	0.15	5.00
16	4.00	0.55	3.50
17	2.00	0.15	5.00
18	4.00	0.30	3.50
19	2.00	0.45	5.00
20	4.00	0.30	3.50

All analytical data were collected in two parallel determinations and the results are presented as mean value (*N*=2), while the results of sensory determinations are presented as panel mean value (*N*=10). Total phenols, antioxidant activity and sensory properties were determined as dependent factors. The response surface modelling (RSM) was used to determine the most suitable models according to the following equation:



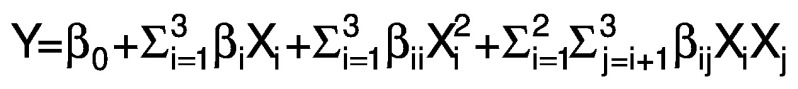



where Y is the predicted result or the value of the dependent variable, β_0_ is the intercept, β_i_, β_ii_ and β_ij_ are the linear, quadratic and interaction coefficients, respectively, and X_i_...X_j_ are the values of the independent variables.

Analysis of variance (ANOVA) was performed to determine the significance of the model and the influence of each factor. A confidence level of 95 % was set for the applied tests. The suitability of the model was tested and verified using several common statistical parameters, *i.e*. by determining the coefficient of determination (R^2^) and the lack of fit. The mentioned dependent variables were also included in the optimisation of the preparation conditions of the fortified almond drink formulation, which was carried out using the desirability method.

## RESULTS AND DISCUSSION

In this study, the almond drink enriched with concentrated aqueous herbal extract of myrtle and bay leaves and fennel seeds and the addition of lecithin as an emulsifier and xylitol as a sweetener was optimised by response surface modelling (RSM). The preliminary study determined the content of each extract in the final concentrated aqueous herbal extract used for the formulations. The blend containing 25 % myrtle, 25 % bay leaf and 50 % fennel extract received the best flavour rating. Although whole milk and other milk alternatives have been similarly fortified (*30*, *31*), there are only few studies on almond drink. Given the high content of bioactive, mainly phenolic compounds in herbal extracts, which have significant antioxidant and antimicrobial activity, enriched products are also expected to have increased bioactivity. Giram *et al.* (*32*) studied the effect of moringa and roselle extracts on the microbiological stability of a product based on cow's milk and soy drink and found that the addition of extracts reduced yeast and mould counts.

### Total phenolic content

The basic almond drink prepared in this study had a TPC of 121.27 mg/L, which is within the range of values reported in the literature. Manzoor *et al.* (*33*) studied the effect of thermosonication of almond drinks and reported a TPC of 702.2 µg/g, while Faraloni *et al.* (*34*) applied hydrodynamic cavitation and obtained up to 140 mg/kg of TPC depending on the applied processing conditions. Similarly, Ceylan (*35*) found TPC in almond drinks in the range of 102–553 mg/kg. On the other hand, Atik *et al.* (*36*) reported only 6.09 mg/kg in the control sample of an almond drink they studied. This wide range of results could be related to the different mass fractions of TPC in the almonds used as raw material, the different preparation methods of the almond drinks in the mentioned studies, but also to the analytical determinations. Namely, the turbidity of the samples occurring during the spectrophotometric measurement can increase the absorbance values and, consequently, the TPC values, which is why the preparations should be filtered before analysis. The TPC values measured in the unfiltered preparations were 670.58 mg/L and decreased significantly after filtration, which we would recommend for determination in milk alternatives and similar products. In addition, the result found in this study agrees with the TPC value of 830.19 mg determined in dry almonds by Banjanin *et al.* (*37*), considering the addition of 10 % (*m*/*V*) ground almonds to the basic almond drink.

The TPC in the fortified almond drink formulations increased linearly with the amount of added concentrated aqueous herbal extract, as shown by the results of ANOVA in Table 2, while the effect of other factors was not detected.

**Table 2:** Experimental data for total phenolic content (TPC), antioxidative activity expressed as Trolox equivalents (TE) (*N*=2) and sensory parameters (*N*=10) in fortified almond drink formulations

Formulation	*γ*(TPC)/(mg/L)	*b*(TE)/(µmol/L)	Sensory property							
Homogeneity	Colour	Herbal odour	Herbal taste	Sweetness	Bitterness	Aftertaste	Overall acceptability
1	52.9	519	5.00	1.70	3.30	3.80	4.40	1.40	2.40	6.80
2	427.1	3342	4.00	4.20	3.20	4.20	4.40	2.20	2.50	5.80
3	462.9	3189	5.00	4.00	2.60	3.10	6.20	1.10	2.30	5.80
4	213.6	2162	4.50	2.60	1.20	1.80	2.20	1.80	1.50	4.40
5	405.7	3365	4.50	4.80	2.70	3.30	1.50	2.70	2.30	4.70
6	707.9	5489	2.00	6.50	4.90	4.50	4.80	3.10	3.30	4.40
7	492.3	5035	2.50	5.50	4.70	5.30	5.70	1.80	2.80	5.70
8	382.9	3962	3.00	4.60	3.10	3.90	4.30	1.30	2.00	6.20
9	573.6	4789	1.00	5.00	3.30	3.80	2.20	2.30	2.40	5.10
10	422.1	3919	3.50	4.30	2.60	3.40	4.80	1.80	1.80	5.20
11	277.9	2277	4.50	3.10	2.10	2.10	2.00	1.50	1.20	5.10
12	647.9	6135	3.00	4.90	4.00	3.90	3.00	2.00	2.00	5.30
13	343.6	3200	3.00	4.10	2.60	3.60	4.50	1.60	1.70	5.00
14	418.6	3042	4.50	3.70	2.90	3.40	4.50	1.40	1.60	5.30
15	623.6	5631	2.00	4.20	4.80	4.10	4.30	2.00	1.90	5.50
16	472.1	3000	3.00	4.40	3.20	3.30	3.40	1.60	1.50	6.10
17	235.7	1765	5.00	2.90	2.30	2.90	3.20	1.70	1.20	5.50
18	446.4	3127	4.50	4.50	2.70	3.10	5.00	1.20	1.40	6.00
19	285.0	1231	4.50	3.20	2.00	2.40	5.00	1.20	1.30	5.70
20	492.1	2958	3.00	4.20	3.50	3.20	2.60	1.80	1.30	5.50

The reduced predictive model equation shown in Table 3 had a coefficient of determination (R^2^) of 0.916, indicating good predictive power of the model, while the lack of fit was not significant (p=0.579).

**Table 3:** Model parameters (regression coefficient, p-value, coefficient of determination (R^2^) and lack of fit) for total phenolic compounds (TPC), antioxidant activity expressed as Trolox equivalents (TE) and sensory properties affected by the amounts of concentrated aqueous herbal extract (A), lecithin (B) and xylitol (C)

Source of variation	*γ*(TPC)/(mg/L)	*b*(TE)(µmol/L)	Sensory property							
Homoge-neity	Colour	Herbal odour	Herbal taste	Sweetness	Bitterness	Aftertaste	Overall acceptability	
Intercept p-value	+38.9 <0.001	+413.3 <0.001	+5.57 <0.001	+1.52 <0.001	+0.58 <0.001	+1.52 0.006	+0.25 <0.001	+1.83 0.005	+3.85 0.024	+4.33 <0.001	
										
A p-value	+88.8 <0.001	+824.2 <0.001	-0.55 <0.001	+0.58 <0.001	+0.44 <0.001	+0.33 <0.001	+0.13 0.272	+0.17 0.004	-0.60 0.003	+0.01 5.659	
										
B p-value	+71.6 0.872	+126.7 0.886	-0.26 0.852	+1.55 0.040	+0.83 0.465	+0.45 0.682	+0.95 0.532	-0.47 0.498	-0.61 0.925	+0.47 0.293	
										
C p-value	+0.9 0.577	-97.5 0.279	+0.09 0.515	-0.06 0.432	+0.15 0.202	+0.13 0.233	+0.82 <0.001	-0.18 0.020	-0.74 0.943	+0.24 <0.001	
										
AB p-value	-	-	-	-	-	-	-	-	+0.29 0.519	-	
										
AC p-value	-	-	-	-	-	-	-	-	+0.02 0.643	-	
										
BC p-value	-	-	-	-	-	-	-	-	+0.94 0.135	-	
										
A^2^ p-value	-	-	-	-	-	-	-	-	+0.08 0.009	-	
										
B^2^ p-value	-	-	-	-	-	-	-	-	-6.33 0.174	-	
										
C^2^ p-value	-	-	-	-	-	-	-	-	+0.05 0.237	-	
										
Lack of fit	0.579	0.365	0.321	0.398	0.088	0.160	0.594	0.504	0.136	0.719	
										
R^2^	0.916	0.910	0.653	0.891	0.649	0.533	0.664	0.541	0.773	0.661	
										
R^2^ adjusted	0.900	0.893	0.588	0.870	0.583	0.445	0.602	0.455	0.569	0.597	

### Antioxidant activity

Similar to TPC, higher amounts of added concentrated aqueous herbal extract also increased the antioxidant activity of the formulations (Table 2 and Table 3). This model showed an even better replication of the observed results with an R^2^ of 0.910, with the lack of fit also not significant (p=0.365). The antioxidant activity of the basic almond drink formulation, expressed as Trolox equivalents, was 410.06 µmol/L, which was lower than the value of 1310 µmol/L determined by Plank *et al.* (*38*). However, in the study by Lipan *et al.* (*39*), the determined antioxidant activity of the almond drink was 47 µmol/L, which is significantly lower than the value determined in this study. The differences in antioxidant activity found in the literature could again be due to the different raw materials used and the different processing conditions for the preparation of almond drinks. Leksawasdi *et al.* (*40*) fortified green soybean milk with the addition of green soybean pod extract and also reported a significant increase in TPC, while other authors reported similar results with different dairy products fortified with herbal extracts (*41*). An increase in TPC from 0.01 to 0.15 g/kg along with an increase in antioxidant capacity from 7.5 to 17.7 (mmol/kg) associated with fortification of whole milk and soy drink with turmeric was also observed by Idowu-Adebayo *et al.* (*42*).

### Sensory properties

The stability and homogeneity of dairy-free milk alternatives are often a challenge in their production, as they are in the form of colloidal systems consisting of small particles dispersed in a medium. These particles can be emulsified lipids, but also parts of crushed plant material containing proteins and carbohydrates, mainly in the form of suspensions (*43*). To stabilise the system of almond drink formulations investigated in this study, lecithin was added as an emulsifier. The data in Table 2 and Table 3 show that sedimentation and decrease in homogeneity of the samples depended on the amount of concentrated aqueous herbal extract added to the formulation, while the amount of lecithin had no effect on the results. The decrease in homogeneity caused by the higher amounts of concentrated aqueous herbal extract could have been due to the increased protein-phenol interactions in the samples with higher TPC. According to Ozdal *et al.* (*44*), secondary and tertiary protein structures are altered by the presence of phenolic compounds and their solubility is reduced, leading to increased sedimentation, although the exact mechanism of these interactions is not fully understood. Considering that the sedimentation of almond drink formulations was caused by the separation of solid particles and proteins, the addition of lecithin as an emulsifier was not sufficient to stabilise them. For this reason, the stability of such products could be improved by adding thickening or gelling hydrocolloids (*e.g.* guar gum, gum arabic, alginate, gelatin, *etc.*). In addition, some of the advanced homogenisation techniques for almond drinks fortified with herbal extracts, such as hydrodynamic cavitation and ultrasonication, could be used to improve stability, as has been done successfully for unfortified almond and hazelnut beverages (*34*, *45*).

As mentioned earlier, almond-based milk alternatives are among the most popular of this group, with a 64 % market share in the United States (*46*). According to Alozie Yetunde and Udofia (*12*), an almond drink has a similar mouthfeel to a soy drink, but is better in terms of colour, flavour, taste and overall acceptability.

**Fig. 2:**
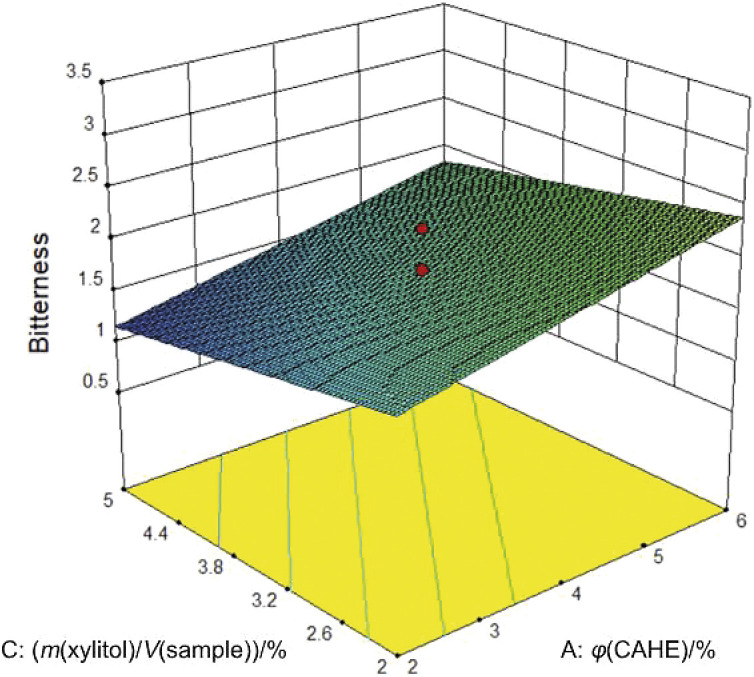
Response surface plot of bitterness as affected by the concentrated aqueous herbal extract (CAHE) (A) and xylitol (C) amount with constant lecithin amount 0.3 % (*m*/*V*). Red dots represent design points above the predicted value, while contour lines indicate the projection of values to two-factor plane

The colour of the samples tested in this study changed depending on the amount of added concentrated aqueous herbal extract and lecithin, as shown in the results of ANOVA and the given model (Table 3). Since the almonds were peeled as part of the processing for the preparation of basic drink, its colour resembled the whitish colour of whole milk. Therefore, the addition of concentrated aqueous herbal extract and lecithin caused the colour of the almond drink formulations to change to darker tones and a latte-like appearance. Similar to the results of this study, Maghsoudlou *et al.* (*4*) also reported an increase in colour intensity detected by the sensory panel of the almond drink samples with higher amounts of rose water. In the work of Kim *et al*. (*47*), the addition of radish oil also increased the colour of dairy and non-dairy drinks, while the increase in colour intensity of milk fortified with tulsi juice, ginger juice and turmeric powder reported by Gaur *et al*. (*48*) was related to an increase in the overall acceptability of the product. As the amount of concentrated aqueous herbal extract increased, the herbal odour and taste of the prepared formulations also increased, with p<0.001 for both properties, with the linear model (Table 3) showing that the average score increased by 0.44 and 0.33 points, respectively, for each percentage of added concentrated aqueous herbal extract. Because of its high aroma acceptability, fennel seed is very often used for different food preparations. This was also confirmed by the preliminary tests conducted for this study, which is why fennel seed extract was added in an amount of 50 % of the concentrated aqueous herbal extract used to make the formulations. Furthermore, in the study by Das *et al*. (*49*), the bread enriched with 5.0 to 7.0 % fennel seed had the best overall acceptability. This is comparable to the high factor level for the addition of *φ*(concentrated aqueous herbal extract)=6 % used in this study. On the other hand, due to the high TPC values of hydroethanolic myrtle and bay leaf extracts demonstrated in a previous study by Cvitković *et al.* (*22*), their extracts were added in a lower amount, *i.e*. 25 % each. However, bitterness was still present, with the higher amounts of concentrated aqueous herbal extract added increasing the bitterness of the formulations (p=0.004). From the linear model obtained, it is obvious that each percent of added concentrated aqueous herbal extract increased the bitterness score by 0.35 (Fig. 2).

On the other hand, bitterness was also affected by the amount of xylitol (p=0.020), which resulted in a decrease in bitterness score of 0.26 per 1 % of added xylitol. The added sweetener was thus able to balance the amount of concentrated aqueous herbal extract and mask its bitterness to some extent. A similar effect was found in the study by Bertelsen *et al.* (*50*), who successfully used xylitol to mask the bitter taste of bioactive protein hydrolysates. Xylitol is not only a natural, low-calorie sweetener that lowers the energy value of formulations, but also has bioactivity that provides benefits for dental health (*51*), relieves constipation and reduces the risk of respiratory infections (*52*). In the study by Shim *et al.* (*53*) of the absorption of phenolic compounds from green tea through the digestive tract, xylitol and citric or ascorbic acid were found to increase the utilisation of catechins threefold and the absorption of catechins in the intestine elevenfold.

**Fig. 3:**
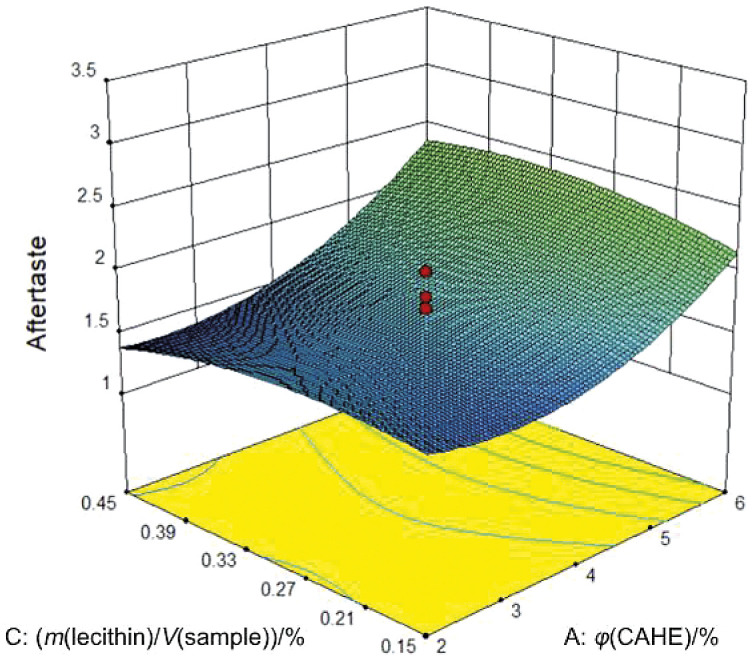
Response surface plot of aftertaste as affected by the concentrated aqueous herbal extract (CAHE) (A) and lecithin (B) amount with constant xylitol amount 3.5 % (*m/V*). Red dots represent design points above the predicted value, while contour lines indicate the projection of values to two- factor plane

The amount of concentrated aqueous herbal extract added to the formulations also contributed to the occurrence of aftertaste, with the model attributing both a linear and quadratic component of this factor to the aftertaste scores with p=0.003 and p=0.009, respectively (Table 3). The response plot in Fig. 3 shows a slight increase in aftertaste values with the addition of up to *φ*(concentrated aqueous herbal extract)=3.5 % and a greater increase at higher volume fractions.

**Fig. 4:**
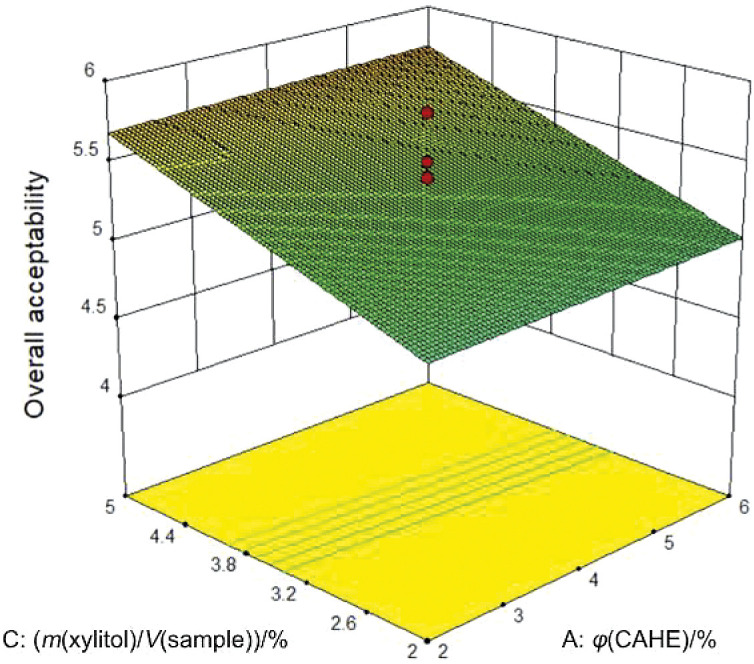
Response surface plot of overall acceptability as affected by the concentrated aqueous herbal extract (CAHE) (A) and xylitol (C) amount with constant lecithin amount 0.3 % (*m*/*V*). Red dots represent design points above the predicted value, while contour lines indicate the projection of values to two factor-plane

Ivanišová *et al.* (*54*) investigated the influence of different herbal extracts on the sensory properties of fortified apple juice and found differences in aftertaste, which was lowest with the addition of lemon balm and thyme and highest with the addition of sage. Belščak-Cvitanović *et al.* (*55*) also observed an aftertaste in chocolate enriched with an extract of raspberry leaves and related it to the herbal flavour of the extract. An increase in aftertaste due to the addition of herbal extracts was also noted by Wihansah *et al.* (*56*), who reported that the addition of fenugreek extract to yoghurt increased the bitter aftertaste, which was unacceptable when more than 2 % of the extract was added. However, in this study, the increase in herbal aroma and aftertaste due to the higher concentrated aqueous herbal extract volume fractions did not result in lower acceptance of the formulations. On the other hand, according to Fig. 4 and model (Table 3), it is evident that the overall acceptability of the samples slightly increases by 0.24 per 1 % (*m*/*V*) of xylitol added, regardless of the addition of plant extracts.

In the study conducted by Santana *et al.* (*57*) on the quality and sensory profile of purified cashew juice, xylitol was also used, and its flavour was positively accepted. All attributes associated with the presence of sugar, such as sweet aroma and very sweet taste, appeared in the opposite quadrant to the unpleasant attributes, indicating the acceptance of sweet products by the sensory panel.

### Optimisation

The optimal formulation was determined by applying the desirability method, which establishes the amounts of ingredients added to the formulation that ensure the best compromise for the desired properties. For this purpose, maximum antioxidant activity, maximum phenolic content and maximum overall acceptability were placed at the 3rd level of importance, while maximum homogeneity, maximum herbal odour, maximum herbal flavour, minimum bitterness and minimum aftertaste were placed at 1st level of importance. Thus, an optimal almond drink formulation with a desirability value of 0.702 was obtained with *φ*(concentrated aqueous herbal extract)=6 %, 0.15 % (*m*/*V*) lecithin and 5 % (*m*/*V*) xylitol. Based on the obtained models, this formulation gave an overall acceptability score of 5.71, while the concentration of total phenols was 572.5 mg/L and the antioxidant activity, expressed in Trolox equivalents, was 5055.07 µmol/L. The values predicted by the application of the models were confirmed by the experimental preparation of the optimal formulation, which gave an overall acceptability score of 5.84, a TPC of 580.1 mg/L and an antioxidant activity of 5103.92 µmol/L.

## CONCLUSIONS

The growing popularity of dairy-free milk alternatives requires the development of functional foods based on ingredients with high nutritional value and bioactivity. All tested almond drink formulations fortified with concentrated aqueous herbal extracts of myrtle and bay leaves and fennel seeds were generally well received by the panellists. However, the amount of added concentrated aqueous herbal extract, xylitol and lecithin affected the results of the analysed chemical and sensory properties. The addition of concentrated aqueous herbal extract increased herbal flavour and odour as well as the aftertaste. However, these properties did not negatively affect overall acceptability, especially in formulations with higher amounts of xylitol, where the potential bitterness of the phenolic compounds was masked by the increased sweetness, which in turn increased overall acceptability. The optimal formulation contained almost 5-fold higher total phenolic content and had more than 12-fold higher antioxidant activity than the basic drink, confirming its functionality. The results of this study confirm the usefulness of the Box-Wilson experimental design and response surface modelling in the characterisation and optimisation of dairy-free milk alternatives, and allow the development of a product with high nutritional value and highly acceptable sensory properties, and thus great potential for commercialisation.
